# Drug Release Properties of Diflunisal from Layer-By-Layer Self-Assembled κ-Carrageenan/Chitosan Nanocapsules: Effect of Deposited Layers

**DOI:** 10.3390/polym10070760

**Published:** 2018-07-10

**Authors:** Sarai Rochín-Wong, Aarón Rosas-Durazo, Paul Zavala-Rivera, Amir Maldonado, María Elisa Martínez-Barbosa, Itziar Vélaz, Judith Tánori

**Affiliations:** 1 Departamento de Investigación en Polímeros y Materiales, Universidad de Sonora, Hermosillo 83000, Sonora, Mexico; sarai.rochin@unison.mx (S.R.-W.); memartinez@polimeros.uson.mx (M.E.M.-B.); 2 Rubio Pharma y Asociados S.A. de C.V., 83210 Hermosillo, Sonora, Mexico; aaron.rosas@rubiopharma.com; 3 Departamento de Ingeniería Química y Metalurgia, Universidad de Sonora, Hermosillo 83000, Sonora, Mexico; paul.zavala@unison.mx; 4 Departamento de Física, Universidad de Sonora, Hermosillo 83000, Sonora, Mexico; maldona@guaymas.uson.mx; 5 Departamento de Química, Facultad de Ciencias, Universidad de Navarra, 31080 Pamplona, Navarra, Spain

**Keywords:** polyelectrolyte, kinetic models, chitosan, carrageenan, layer-by-layer, self-assembly, diflunisal, release mechanisms, nanoemulsion

## Abstract

Engineering of multifunctional drug nanocarriers combining stability and good release properties remains a great challenge. In this work, natural polymers κ-carrageenan (κ-CAR) and chitosan (CS) were deposited onto olive oil nanoemulsion droplets (NE) via layer-by-layer (LbL) self-assembly to study the release mechanisms of the anti-inflammatory diflunisal (DF) as a lipophilic drug model. The nano-systems were characterized by dynamic light scattering (DLS), zeta potential (ζ-potential) measurements, transmission electron microscopy (TEM), scanning electron microscopy (SEM), X-ray energy dispersive spectroscopy (XEDS) and Fourier transform infrared spectroscopy (FTIR) to confirm the NE-coating with polymer layers. In addition, kinetic release studies of DF were developed by the dialysis diffusion bag technique. Mathematical models were applied to investigate the release mechanisms. The results showed that stable and suitably sized nanocapsules (~300 nm) were formed. Also, the consecutive adsorption of polyelectrolytes by charge reversal was evidenced. More interestingly, the drug release mechanism varied depending on the number of layers deposited. The nanosized systems containing up to two layers showed anomalous transport and first order kinetics. Formulations with three and four layers exhibited Case II transport releasing diflunisal with zero order kinetics. Hence, our results suggest that these polyelectrolyte nanocapsules have great potential as a multifunctional nanocarrier for drug delivery applications.

## 1. Introduction

In recent years, interest in the field of active compounds release has increased. The result has been the development of innovative drug nanocarriers to enhance drug bioavailability, protect the molecules from the biological environment, and provide sustained release. This has great potential for multi-compartmental delivery devices [1]. Among the materials that have been proposed, biopolymer multilayer capsules are especially relevant to a wide range of fields such as medicine, pharmaceutics, food packing, and cosmetology [2,3,4].

In the field of drug delivery, several polymers are recognized as promising materials for the preparation of nanocarriers. They functionalize surfaces, acting as a polyelectrolyte coating for different templates such as spheres or films [5,6,7]. Taking advantage of the self-assembly properties of the polyelectrolytes, they can form multilayer polymeric structures via a layer-by-layer technique (LbL), which consists of the alternate deposition of oppositely charged materials, mainly polyelectrolytes, on to templates of different size and composition through electrostatic interactions [1,4].

Many of the recently studied polyelectrolytes for drug release are of synthetic origin [8,9,10]. However, the increasing demand for product biocompatibility and biodegradability supports consideration of natural ones [11]. Some natural polymers such as carrageenan and chitosan have been studied extensively for drug delivery applications [6,7,12,13] and templates for tissue engineering [14]. These polymers present better biocompatibility and biodegradability than the commonly used synthetic polymeric materials [15].

Multilayer films can be used as masks or patches on skin and organs for controlled release of certain active substances [14,16]. However, their micrometric size limits them from reacting with other biological structures [17]. Similarly, the templates used for the synthesis of core-shell particles are also often found in the range of microns [9,10,18], which restricts their use in the field of drug release. In recent years, the synthesis of hollow polymer nanocapsules has been reported [5,17,19,20]. Furthermore, by using natural and biocompatible polymers such as chitosan and carrageenan for their preparation it is possible to obtain hollow polymer capsules at the nanometric scale. Despite these achievements, the hollow polymer nanocapsules are limited only to encapsulate hydrophilic compounds. In addition, they have the disadvantage that during their synthesis, acids must be used to dissolve the cores or templates and thus the polymer shell may collapse [10,21], therefore, it is often necessary to resort to additional steps of cross-linking the polymer to increase mechanical strength [17]. To overcome these limitations, great interest has developed in formulations based on nanoemulsion-templated layer-by-layer (LbL) capsules [22] because of their ability to act as carrier systems for both oil and water-soluble functional compounds.

The classical LbL approach involves the alternative deposition of polyelectrolytes onto colloidal templates in aqueous media with intermediate centrifugation/redispersion cycles to ensure removal of any unadsorbed polyelectrolyte. In recent years, some authors have proposed various modifications to the LbL fabrication of nanocapsules [23], for example, the use of semi-continuous membrane filtration [24], immobilizing the core particles in a gel film [25], or using fluidic assembly approach [26,27]. However, some of these procedures require constant control, specialized equipment, and in some cases, they produce a relatively small number of capsules. Recently, Elizarova and Luckham [21] proposed the use of a tubular flow type reactor for continuous fabrication of capsules, but this process is also limited because the excess polyelectrolyte is not completely removed after each deposition cycle [4]. Among the various methods proposed, the classical LbL approach is still attractive because the intermediate centrifugation/redispersion steps remove the excess non-adsorbed PE; as a result, avoiding the formation of interpolyelectrolyte complexes in the media and allowing the adsorption of the following PE of opposite charge.

On the other hand, one common goal of a controlled-release matrix system is to prevent the fluctuation of the therapeutic concentration of the drug in the body and thus decrease the possible side effects that may occur. This can be achieved by exerting control over the duration and rate of drug release which depends on the mechanisms implicated [28]. Most of the previous studies are limited to studying the release profiles [5,13,15,29] and in some cases the kinetic data are adjusted to a single release model [1]. However, knowing the mechanisms of molecular release from these devices can be very helpful in the design and evaluation of their utility as drug delivery systems in pharmaceutical applications.

**Figure 1:**
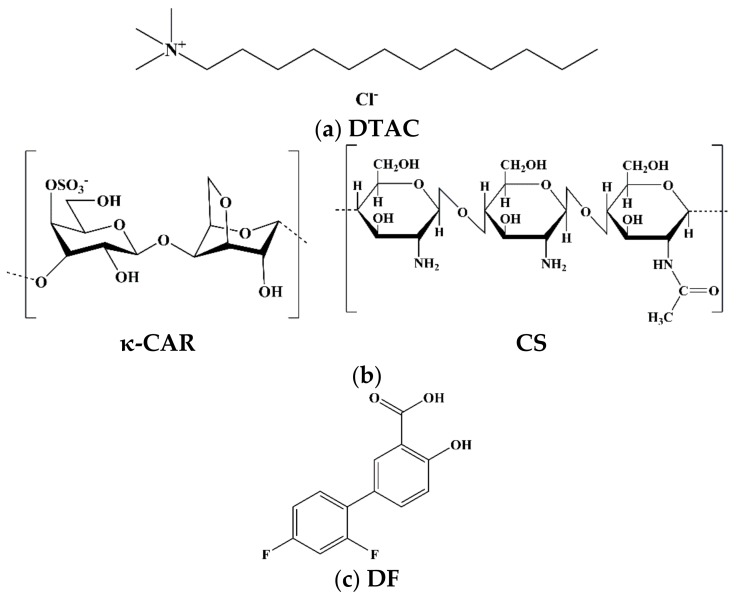
Chemical structures of the nanocapsule compounds: (**a**) surfactant; (**b**) polyelectrolytes (PE); and (**c**) diflunisal (DF).

The aim of this paper is to report the formulation, characterization, and drug release properties of biocompatible multilayer nanocapsules based on a nanoemulsion template which is stabilized by dodecyltrimethylammonium chloride (DTAC). The synthesis of LbL polyelectrolyte nanocapsules of κ-carrageenan as polyanion and chitosan as polycation is proposed due to the ability of the carrageenan sulphate groups to associate with chitosan protonated amino groups via electrostatic interactions. This results in the formation of a multilayer polyelectrolyte nanocapsule by the LbL self-assembly approach. This synthesis avoids the use of acids and specialized equipment. All of the procedure is carried out in an aqueous system and only one centrifugation/redispersion cycle is required. More importantly, we study the drug release behavior encapsulating diflunisal (DF) as a small lipophilic drug model (DF is mainly hydrophobic but has a solubility of 14.5 mg·mL^−1^ in alkaline aqueous solutions). The results were adjusted to kinetic models to determine the predominant release mechanisms as a function of the number of deposited layers to discuss their applicability as controlled release systems. The molecular structures of DTAC, κ-carrageenan, chitosan, and diflunisal are shown in Figure 1.

## 2. Materials and Methods

### 2.1. Materials

κ-Carrageenan (κ-CAR) was provided by Sigma Aldrich, Co., St. Louis, MO, USA (Lot No. SLBH9868V). According to the supplier, this sample contains 11% K, 2.0% Na and 3.5% Ca. Low molecular weight chitosan (CS; SLBH5374V) with 75% deacetylation was also obtained from Sigma Aldrich, Co., St. Louis, MO, USA. Both polymers were used without further purification. Dodecyltrimethylammonium chloride (DTAC) was obtained from Aldrich Chemistry, Steinheim, DE. Olive oil was supplied by Fluka^®^, St. Louis, MO, USA. Acetone was purchased from VETEC, St. Louis, MO, USA and Ethyl alcohol from FagaLab, Mocorito, MEX. Diflunisal (DF; 2′,4′-difluoro-4-hydroxy-(1,1′-biphenyl)-3-carboxylic acid) was supplied by Merck Sharp and Dohme, Madrid, ESP. Release media were prepared using the following reagents: HCl and Na_2_HPO_4_ (99%) from Sigma Aldrich and KH_2_PO_4_ (99%) from Panreac, Barcelona, ESP. All other reagents were of analytical grade without further purification. Milli-Q grade water (18.2 MΩ.cm at 25 °C) was used throughout the procedures.

### 2.2. Formulation of Nanoemulsions (NE)

Nanoemulsions were prepared according to the general procedure described by Rosas-Durazo et al. [30] adapted to a different oil component in our system. It was prepared by dissolving 120 mg of DTAC in 0.5 mL of ethanol, 125 µL of olive oil (containing or not 25 mg·mL^−1^ of DF) and 9.5 mL of acetone. Immediately afterwards, this organic solution was poured under moderate magnetic stirring (100 rpm; Cimarec Basic, Thermoscientific, Waltham, MA, USA), into 20 mL of water. The mixture immediately turned milky as a result of the spontaneous formation of the emulsion nanodroplets, due to the diffusion of acetone and ethanol to the aqueous phase. Subsequently, acetone, ethanol, and a portion of water were evaporated in a rotavapor at 40 °C (~ 8 min, 100 rpm, 10 kPa) to a final volume corresponding with one third of the original one (~10 mL), producing an aqueous nanoemulsion.

### 2.3. Preparation of NE(κ-CAR/CS)_x_ Nanocapsules by Layer-By-Layer Self-Assembly

For the LbL build-up, we proceeded by alternating polyelectrolyte deposition starting with the polyanion κ-CAR. Aliquots of the NE (125 µL), prepared as mentioned before, were incubated with an aqueous solution of κ-CAR (20 µL; 0.5 mg·mL^−1^) at 25 °C in a 96-well plate (Corning, New York, NY, USA) mixed lightly and left standing for at least 1 h. After that, the excess of unadsorbed polyelectrolyte was removed by centrifugation (23,000× *g*, 1 h, 15 °C) to minimize the formation of aggregates. This procedure was repeated, this time using a 2% acetic acid CS solution (0.5 mg·mL^−1^) as cationic polyelectrolyte. The alternate deposition of κ-CAR and CS was repeated until the desired layers were attained. A concentration of 1 mg·mL^−1^ of both polyelectrolytes was used in the following layers. The layers of κ-CAR and CS coating were defined as a bilayer and denoted as (κ-CAR/CS)*_x_*.

### 2.4. Characterization of NE and NE(κ-CAR/CS)_x_ Nanocapsules

#### 2.4.1. Particle-Size and Zeta Potential Measurements

The size and polydispersity (PdI) of the systems were determined by using a Zetasizer Nano ZSP (Malvern Instruments, Inc., Worcestershire, UK) by the technique of dynamic light scattering (DLS; detection angle of 173° and fitted with a red laser light, λ = 633 nm). Each sample was analyzed in a polystyrene cell (DTS0012; Malvern Instruments, Inc., Worcestershire, UK). The surface charge was investigated by measuring ζ-potential (zeta potential) on the same instrument, using capillary cells (DTS1070; Malvern Instruments, Inc., Worcestershire, UK). Both measurements were performed at 25 °C and with 1:100 diluted samples due to the opaque optical properties of the dispersions.

#### 2.4.2. Morphological Analyses

Transmission electron microscopy (TEM) and scanning electron microscopy (SEM) analyses were performed to obtain images of both the NE and the oil-core nanocapsules NE(κ-CAR/CS)_2_. The morphology and size distribution of selected systems were determined by using a JEM 2010F transmission electron microscope with operating voltage of 200 kV (JEOL, Ltd., Tokyo, Japan). For TEM observations of NE and NE(κ-CAR/CS)_2_, a drop of the sample solution was spread on the smooth film of the carbon-coated copper TEM grid and dried. After that a drop of 0.5% phosphotungstic acid was spread on the TEM grid for 1 min and the excess was absorbed by touching the edge of the grid with filter paper. All samples were subsequently dried in vacuum before observation. The characteristic chemical elements of the NE and oil-core nanocapsules were identified by XEDS analysis (Quantax 200 X-ray energy dispersive spectrometer, Bruker, GmbH, Berlin, Germany). Also, the morphology was probed using a Pemtron SS-300LV scanning electron microscope (Pemtron, Corp., Seoul, Korea). To obtain SEM images, samples of NE and nanocapsule suspensions were deposited on glass slides and dried. After being dried, the samples were coated (Sputter Coater 108 Cressington self Ted Pella Inc., Redding, CA, USA) with a thin gold layer (thickness of about 4 nm).

#### 2.4.3. Fourier Transform Infrared (FTIR) Spectroscopy

To confirm the presence of biopolymers in the nanocapsules, FTIR analyses were carried out with a Fourier-transform infrared spectrometer (Frontier, Perkin Elmer, Waltham, MA, USA). For each sample 32 scans were acquired at a spectral resolution of 1 cm^−1^ in a wavenumber region of 600–4000 cm^−1^. The NE and oil-core nanocapsules were embedded in KBr, dried at 100 °C for 5 h, and pressed into 1 mm thick pellets for FTIR measurements.

### 2.5. Drug Release

#### 2.5.1. Encapsulation Efficiency

Diflunisal-loaded nanoemulsions were prepared as mentioned in Section 2.2. After loading, the DF-loaded NE was separated from the non-encapsulated DF by ultracentrifugation for 60 min at 23,000× *g* and 15 °C. In order to calculate the DF amount encapsulated, the fluorescence intensity (F900, Edinburgh Instruments, Livingston, UK; λ excitation/emission: 258/420 nm, slits: 3 nm) of free DF in solution after centrifugation was measured. Previously, a calibration curve was obtained. The encapsulation efficiency (EE%) was calculated using Equation (1),

EE% = (DF_total_ − DF_free_/DF_total_) × 100 (1)

where DF_total_ is the initial amount of diflunisal, DF_free_ is the amount of non-encapsulated diflunisal [31]. DF-loaded NE was coated after loading with different layers of polyelectrolytes according to the previously described methodology (see Section 2.3).

#### 2.5.2. In Vitro Drug Release

The in vitro DF release kinetics were performed by the dialysis bag diffusion technique. A suspension (3 mL) of DF-loaded nanoemulsion or DF-loaded nanocapsules was added into a dialysis membrane (molecular weight cut-off 6–8 kDa; Spectra/Por 1, Spectrum Laboratories, Inc., Rancho Domínguez, CA, USA) that was subsequently placed into the release media (50 mL of phosphate buffer pH 7.4 and HCl pH 1.2) under moderate magnetic stirring (125 rpm) for 480 min at 37 ± 0.2 °C. At appropriate time intervals, aliquots of 1 mL of release media were taken and were replaced with fresh buffer. The amount of diflunisal released from the systems was evaluated by measuring the emission fluorescence at 420 nm (F900, Edinburgh Instruments, Livingston, UK). All experiments were performed in triplicate.

### 2.6. Mathematical Modelling

In order to determine the release mechanism of diflunisal and to compare the effect of the number of layers in the nanocapsules on the release profile, we have fitted the release data to some models available in the literature. These models describe the initial rate of drug release, depending on the physical mechanisms that contribute to the nanocapsule unloading. The two main mechanisms are related to Fickian diffusion and to relaxation processes of the polymer chains. All the models quantify the evolution of the amount of drug released as a function of time: *M_t_* = *M_t_*(*t*). The release rate, *r*, is given by the time derivate of *M_t_*: *r* = $\frac{dM_{t}\left(t\right)}{dt}$. We have used the following models.

#### 2.6.1. Korsmeyer-Peppas Model

The Korsmeyer–Peppas equation describes the general solute release behavior from different polymeric devices. In this model, drug release is related with the elapsed time by the equation [32,33]:

(2) $$\frac{M_{t}}{M_{\infty }}=k_{KP}t^{n}$$

where $M_{t}$/$M_{\infty }$ is the fractional amount of the drug released at time *t* (min), *n* is a diffusion exponent indicating the release mechanism, and $k_{KP}$ is a characteristic constant of the system. $M_{\infty }$ is the total drug released (*t* = ∞). It is well established that values of *n* around 0.5 are found when the release mechanism is Fickian diffusion. On the other hand, values of *n* in the range 0.5–1.0 indicate anomalous non-Fickian transport; a value of *n* = 1.0 means Case II transport (zero order) [28,32]. Note that the release rate is proportional to $k_{KP}$ and *n*: $r\propto nk_{KP}t^{n-1}$.

#### 2.6.2. Higuchi Model

A simplified Higuchi model can be expressed as [34]:

(3) $$\frac{M_{t}}{M_{\infty }}=k_{H}t^{0.5}$$

where $M_{t}$/$M_{\infty }$ is the fractional amount of the drug released at time *t* (min), and *k_H_* is the Higuchi diffusion constant. Profiles which have simple Fickian diffusion as the predominant mechanism show good correlation with this model.

#### 2.6.3. First Order Kinetic Model

The first order equation can be expressed as [35]:

(4) $$\frac{M_{t}}{M_{\infty }}=1-e^{-k_{1}t}$$

where $M_{t}$/$M_{\infty }$ is the fractional amount of the drug released at time *t* (min), and *k_1_* is the first order release constant. This model adequately describes the release profiles when the predominant mechanism is related with anomalous diffusion.

#### 2.6.4. Zero Order Kinetic Model

The equation for zero order drug delivery is expressed [36]:

(5) $$\frac{M_{t}}{M_{\infty }}=k_{0}t$$

where $M_{t}$/$M_{\infty }$ is the fractional amount of the drug released at time *t* (min), and *k*_0_ is the zero order release constant. The release profiles that present a better fit to this model generally agree with those that show a Case II transport mechanism in the Korsmeyer–Peppas model. Note that in this model, the release rate is constant: *r* = *k*_0_∙*M*_∞_.

We have applied these models to our release data, fitting the first points (from 0 to 60% of the total drug released) [37] in each curve in order to have an idea of the release mechanism.

### 2.7. Statistical Analysis

All data are expressed as mean ± standard deviation (*n* = 3). The differences between groups were assessed with a one-way ANOVA with Tuckey’s post hoc test. A value of *p* < 0.01 was considered statistically significant.

## 3. Results and Discussion

### 3.1. Characterization of NE and NE(κ-CAR/CS)_x_ Oil-Core Nanocapsules

#### 3.1.1. Particle Size

**Figure 2:**
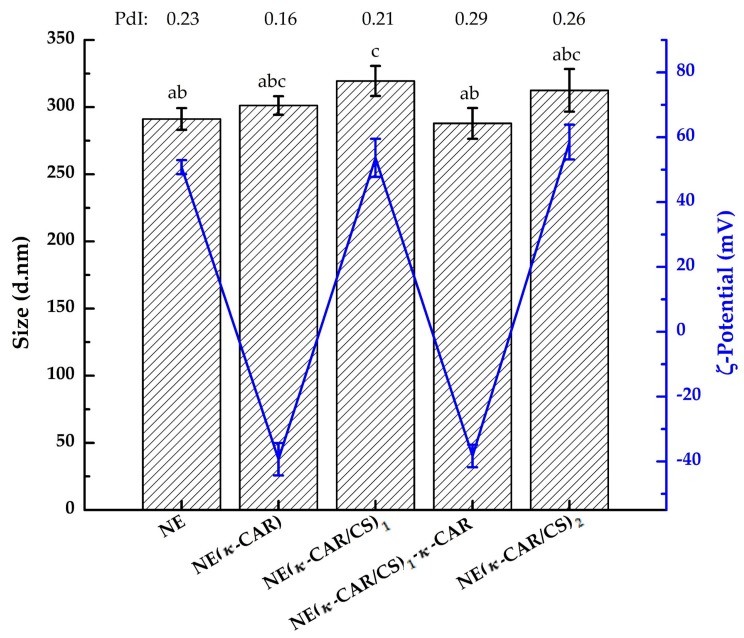
Average hydrodynamic diameter (column), polydispersity index (PdI), and ζ-potential values (line) of NE(κ-CAR/CS)*_x_* oil-core nanocapsules, determined by DLS as function of the number of deposited polyelectrolyte layers. Standard deviation is indicated (*n* = 3). Different letters (a, b, c) indicate significant differences in average hydrodynamic diameter (*p* < 0.01).

As shown in Figure 2, the mean diameter of the NE droplets was 291.2 ± 8.1 nm (zero polyelectrolyte layers) whereas after the deposition of κ-CAR the diameter was 301.2 ± 6.9 nm. The average diameter of the nanocapsules with two layers [NE(κ-CAR/CS)_1_] increased to 319.5 ± 11.2 nm while for the nanocapsules with four layers [NE(κ-CAR/CS)_2_] it was 312.5 ± 15.9 nm. The size of the NE(κ-CAR/CS)_2_ showed an increase of ~21 nm with the adsorption of two bilayers over the initial NE. In general, the values reported for the thickness of the bilayer assembled under standard conditions range between 3 and 7 nm [38]. In this case, the increase of 21 nm for two polymeric bilayers was higher than the average reported, this is probably due to the fact that these values depend on the conditions of the multilayer build-up (pH, salt, organic solvent, etc.) and the nature of the polyelectrolytes used.

An interesting phenomenon is also shown in Figure 2. After the deposition of the first two layers, the following deposition of κ-CAR (3rd layer) resulted in a significant decrease in the nanocapsule size. Liu et al. [17] observed the same trend of decrease in size when depositing ι-carrageenan (iota-carrageenan) after each bilayer of SiO_2_(ι-CAR/CS)*_x_* nanospheres. They attributed that outcome to the difference in charge density of both polymers. Values of PdI (Figure 2) obtained by DLS measurements were used for analyzing the sample quality. The PdI in the range above 0.5 indicates samples of a poor quality due to the high size polydispersity of the particles [39]. For the obtained oil-core nanocapsules with the PE multilayer shells the PdI values were less than 0.3 (Figure 2) which indicate a narrow size distribution and the good quality of the samples. On the other hand, capsules in the range of 100–300 nm are the most suitable for drug delivery applications [40] due to their unique interactions with biological systems at mesoscopic level [41]. Thus, the prepared capsules in our work, have a size in the range suitable for pharmaceutical applications.

#### 3.1.2. Zeta Potential Measurements

κ-CAR and CS adsorption was followed by inspection of the surface charge of the solution systems after the addition of each polymer layer. The initial NE droplets (zero layers) presented positive charge on its surface due to the quaternary ammonium of the DTAC cationic surfactant (Figure 2). Moreover, the ζ-potential of the nanocapsules changed alternatively from negative to positive. The negative values correspond to κ-CAR layers and the positive values correspond to the CS layers, indicating successful coating after each polyelectrolyte deposited layer. In addition, Figure 2 shows ζ-potential values of less than −30 mV or more than +45 mV when the outer layer was κ-CAR or CS, respectively. These results showed that the oil-core nanocapsules suspensions were stable due to strong electrostatic repulsive forces between them [17,39].

#### 3.1.3. Morphological Analyses

**Figure 3:**
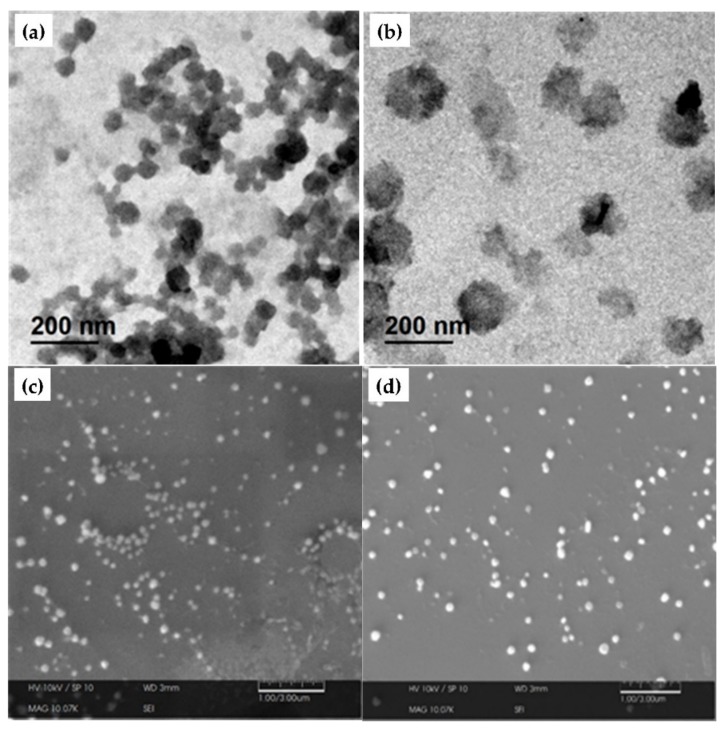
TEM images of (**a**) NE and (**b**) NE(κ-CAR/CS)_2_. SEM images of (**c**) NE and (**d**) NE(κ-CAR/CS)_2_.

TEM and SEM analyses were performed to obtain images, of both, the NE and the NE(κ-CAR/CS)_2_ oil-core nanocapsules. The results of the observed systems are shown in Figure 3 where the morphology and size of NE ((a) TEM and (c) SEM) and NE(κ-CAR/CS)_2_ nanocapsules ((b) TEM and (d) SEM) can be seen. In general, smaller spherical particles are observed compared to the hydrodynamic diameter obtained by photon correlation spectroscopy (Figure 2). This contraction in size is probably due to the difference in the sample state [42]. The size of hydrated layers on the NE surface may increase the value of the average hydrodynamic diameter during DLS analysis [43]. On the other hand, TEM and SEM provide the droplet images in a dry state, thus, smaller droplet sizes were observed. Similar results were reported by Kittitheeranun et al. [44]. Additional atomic force microscopy (AFM) images are available in the Supplementary Materials, Section 2.1.

#### 3.1.4. X-ray Energy Dispersive Spectrometry (XEDS) Analysis

**Figure 4:**
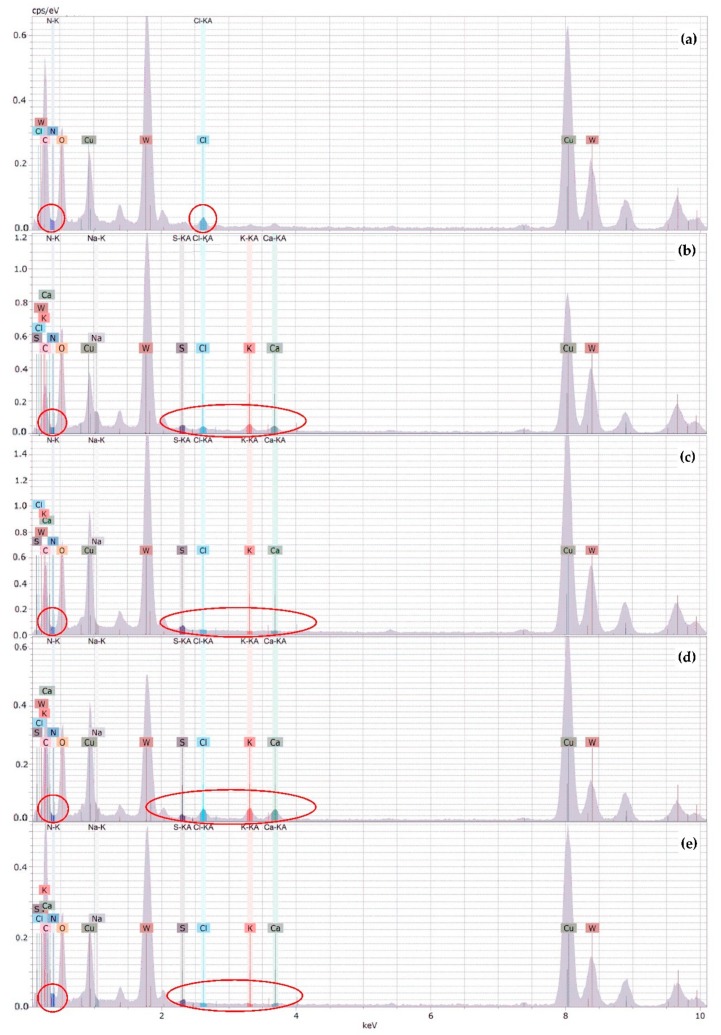
XEDS spectra of (**a**) NE; (**b**) NE-κ-CAR; (**c**) NE(κ-CAR/CS)_1_; (**d**) NE(κ-CAR/CS)_1_-κ-CAR; and (**e**) NE(κ-CAR/CS)_2_.

XEDS analyses were carried out to qualitatively examine the content variation of characteristic elements of DTAC (N and Cl), κ-CAR (S, K, Ca, and Na), and CS (N) in NE and oil-core nanocapsules. Figure 4 shows spectral lines of the XEDS for (a) NE, (b) NE-κ-CAR, (c) NE(κ-CAR/CS)_1_, (d) NE(κ-CAR/CS)_1_-κ-CAR, and (e) NE(κ-CAR/CS)_2_ corresponding to the main elements present in the sample. In the NE spectra (Figure 4a), N and Cl are the elements present because of the DTAC surfactant. Both NE-κ-CAR and NE(κ-CAR/CS)_1_-κ-CAR (Figure 4b,d) had a high content of characteristic elements of κ-CAR: S, Na, K, and Ca while the proportion of N increases in NE(κ-CAR/CS)_1_ and NE(κ-CAR/CS)_2_ (Figure 4b,e), but the proportion of S, Na, K, and Ca decreases. The presence of tungsten (W) in all samples is due to the phosphotungstic acid staining in the sample and the peaks of Cu derive from the Cu grid.

#### 3.1.5. Fourier Transform Infrared (FTIR) Spectroscopy

**Figure 5:**
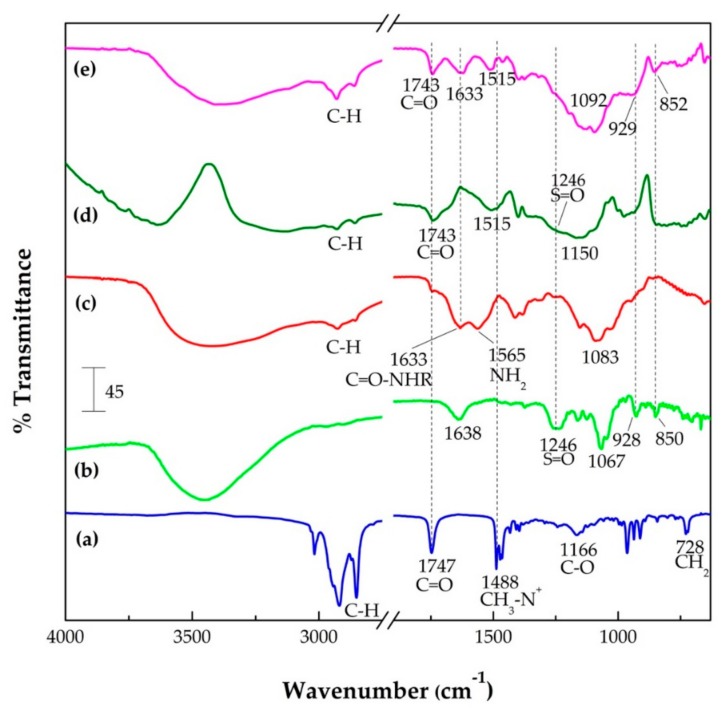
FTIR spectra of: (**a**) NE; (**b**) κ-CAR; (**c**) CS; (**d**) NE-κ-CAR; and (**e**) NE(κ-CAR/CS)_2_.

In order to obtain evidence of the presence of the characteristic functional groups of the components of the NE and oil-core nanocapsules, analyses by FTIR spectroscopy were carried out. Figure 5 shows the FTIR spectra obtained from the analysis of a sample of NE, κ-CAR and CS polymers, as well as samples of oil-core nanocapsules: NE(κ-CAR) and NE(κ-CAR/CS)_2_. The FTIR spectra of κ-carrageenan (Figure 5b) shows a signal at 1246 cm^−1^ corresponding to the sulfate groups, one peak around 1067 cm^−1^ attributed to the glycosidic bond, a peak at 928 cm^−1^ which corresponds to 3,6-anhydrogalactose, and another at 850 cm^−1^, corresponding to galactose-4-sulfate. These results are in accord to the ones reported in the literature [14,45]. In the chitosan spectra (Figure 5c), typical signals at 1633 and 1565 cm^−1^ corresponding to carbonyl groups (C=O–NHR) and amines (NH_2_), respectively, are shown [14]. Additionally, a peak corresponding to glycosidic bonds at 1083 cm^−1^ was identified [46,47].

In the FTIR spectra obtained from nanocapsules of κ-carrageenan (Figure 5d), we can see some of the NE’s own signals. Weak signals around 2900 cm^−1^ correspond to the C–H stretch vibration of both olive oil and the hydrocarbon chain of DTAC surfactant. The band at 1743 cm^−1^ corresponds to the C=O groups of olive oil triglycerides. Likewise, the signal at 1515 cm^−1^ can be attributed to the CH_3_–N^+^ groups of DTAC. On the other hand, the typical band of the sulfate group of carrageenan was unified in a single broad band together with the region of the glycosidic bonds [48]; however, the signals of 3,6-anhydrogalactose and galactose-4-sulfate (928 and 850 cm^−1^, respectively) could not be evidenced as individual signals. In the case of the FTIR spectra of nanocapsules with two κ-CAR/CS bilayers (Figure 5e), the typical absorption bands corresponding to carbonyl groups (C=O–NHR) and amines (NH_2_) of chitosan became a single band at 1633 cm^−1^, these results are in agreement with the literature [46]. In addition, the band around 1388 cm^−1^ also belongs to the cationic polymer (symmetric deformation of CH_3_). With respect to κ-CAR, the signals corresponding to 3,6-anhydrogalactose and galactose-4-sulfate (929 and 852 cm^−1^, respectively) can be appreciated. Note that the main interactions in our system are electrostatic, nevertheless, we also have hydrophobic forces that promote the formation of the nanoemulsion with the interactions between the DTAC and the triglycerides of the olive oil, and H-bonding between the polyelectrolytes [46,49]. Since DTAC is a cationic surfactant, the nanoemulsion droplets bear positive charge. When κ-carrageenan is added to the system, this molecule binds to the droplets due to the negative charge in its OSO_3_^−^ group. When chitosan is added, its NH_3_^+^ group is electrostatically attracted to κ-carrageenan. These electrostatic interactions promote the formation of polyelectrolyte layers around the nanoemulsion droplets. Also, these interactions could be responsible for the shifts in the C=O and CH_3_–N^+^ bands. As observed in Figure 5, the C=O band, due to the triglycerides in olive oil, is located at 1747 cm^−1^ and, the CH_3_–N^+^ band due to the DTAC polar head is located at 1488 cm^−1^, for the bare nanoemulsion (Figure 5a). With the first κ-carrageenan layer, these peaks shift to 1743 and 1515 cm^−1^, respectively (Figure 5d). In the case of the CH_3_–N^+^ band, the shift is probably due to the electrostatic interactions. For the C=O bonds of the triglyceride molecules, the modification of its vibration is probably due to the DTAC-κ-carrageenan interaction. The triglyceride molecules are located parallel to the DTAC hydrophobic tails [50], thus the modifications in the conformation of DTAC (due to the binding of κ-carrageenan) influence the triglyceride vibrations. When chitosan is added to the system, the peak does not shift (Figure 5e), meaning that the main effect is due to the interaction of κ-carrageenan and DTAC.

### 3.2. In Vitro Drug Release

Oil core κ-CAR/CS nanocapsules present per se bioactive potential due to the properties of the olive oil and the biopolymers; however, to study the release properties of a hydrophobic drug, diflunisal was pre-encapsulated into oil droplets NE and later coated with κ-CAR/CS polyelectrolyte layers. The release of the drug was analyzed from samples with zero, one, two, three, and four polyelectrolyte layers. The encapsulation efficiency (EE%) is an important parameter for assessing the capacity of the capsules to retain DF. Samples were analyzed in triplicate, and it was found that 72.3 ± 5.3% of the total DF was encapsulated into the nanoemulsion system. EE% is related to the high lipophilicity of the drug, and it can be concluded that the system is adequate for encapsulation of hydrophobic actives. The presence of the drug in the NE was also observed in the FT-IR spectra for nanoemulsion loaded with diflunisal (see Section 2.2 of the Supplementary Materials).

**Figure 6:**
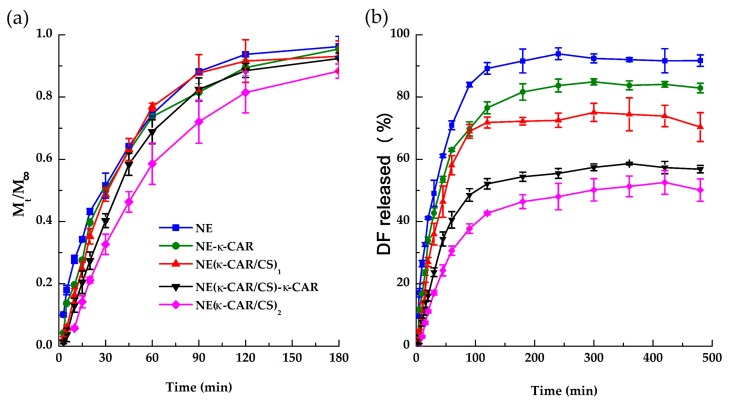
DF cumulative released at pH 7.4 and 37 °C from nanoemulsion (NE) and nanocapsules with one (NE-κ-CAR), two [NE(κ-CAR/CS)_1_], three [NE(κ-CAR/CS)_1_-κ-CAR], and four [NE(κ-CAR/CS)_2_] PE layers, expressed as (**a**) M_t_/M_∞_ and (**b**) percentage released. Standard deviation is indicated (*n* = 3).

Figure 6a shows the time-dependent release profiles of DF from the polyelectrolyte-free nanoemulsion (NE) and from oil-core nanocapsules with one, two, three, and four polyelectrolyte layers at pH 7.4 and 37 °C. The amount released (M_t_) is expressed as a fraction related to the total amount released (M_∞_). Comparing the obtained release profiles, it is evident that the presence of polyelectrolyte shell offers a barrier for the release of the loaded diflunisal. In fact, after the first 60 min the amount of DF released from NE, NE(κ-CAR), and NE(κ-CAR/CS)_1_ is almost 80%. However, in the case of NE(κ-CAR/CS)_2_ nanocapsules, the same amount of DF is released in 120 min.

Figure 6b shows the percentage of DF released from the systems at pH 7.4 and 37 °C. Approximately 95% of the encapsulated DF was released from NE. On the other hand, nanocapsules with one (NE-κ-CAR) and two layers [NE(κ-CAR/CS)_1_] delivered 85 and 78%, respectively; meanwhile, only 59 and 53% was released from nanocapsules with three [NE(κ-CAR/CS)_1_-κ-CAR] and four layers [NE(κ-CAR/CS)_2_], respectively. It is worth mentioning that, at pH 1.2, DF was not released from any of the systems, or at least it was not detectable by spectrofluorimetry analysis. These results suggest that the DF delivery from the oil core κ-CAR/CS nanocapsules is pH sensitive, and this lipophilic drug might be released in gastrointestinal medium and the blood system rather than in the acidic stomach [51]. The solubility of DF likely influences the release kinetics. Diflunisal is practically insoluble in aqueous conditions (14.5 mg·L^−1^); the drug (pKa = 2.94) exists in its neutral form in pH 1.2 while in pH 7.4 the drug is in its anionic form, with higher solubility.

#### Mathematical Modelling

In order to more precisely investigate the DF release mechanism from the oil-core nanocapsules, the experimental data were analyzed by applying the models described in Section 2.6, and the results are reported in Table 1. We obtained the value of the exponent *n* for all the studied samples using the Korsmeyer-Peppas equation. As can be seen in the grey zone of Table 1, the obtained *n* values suggest different transport mechanisms. In all cases, good coefficients of determination were obtained.

The *n* value obtained for the polyelectrolyte-free nanoemulsion (NE) is 0.56 ± 0.02, suggesting a Fickian transport mechanism (expected value around *n* = 0.5). This result was further confirmed by fitting the NE data to the Higuchi model Equation (3) typical of Fickian diffusion. As it can be appreciated in Table 1, this fit has a better correlation for this sample than the first and zero order models.

Referring to the nanocapsules, the values of *n* are higher than those of NE. For NE(κ-CAR) (one polyelectrolyte layer) and NE(κ-CAR/CS)_1_ (two polyelectrolyte layers) *n* was determined to be 0.74 ± 0.01 and 0.89 ± 0.01, respectively (Table 1). These data display an acceptable fitting to the first order kinetic model Equation (4), indicating an anomalous non-Fickian transport mechanism (mixed diffusion and relaxation mechanism). The transition from the Fickian to the non-Fickian mechanism is due to the presence of the polyelectrolyte layers added to the nanoemulsion droplets. The drug release occurs in a single step, without burst effect. The absence of burst releases indicates that all the drug is encapsulated in concordance with the encapsulation efficiency value [52]. Note that the release constants values are similar.

Moreover, the *n* values for the NE(κ-CAR/CS)_1_-κ-CAR (three layers) and NE(κ-CAR/CS)_2_ (four layers) systems are very close to 1 (see Table 1), suggesting a Case II transport mechanism in the Korsmeyer-Peppas model. As expected, these data display a good correlation with the zero order model Equation (5), especially for the three layers system. Thus, in these two systems the diflunisal release rate is constant which is often a desired property of a controlled release device because it enhances therapeutic efficacy and minimizes toxic effects [53,54].

Note that in Table 1 we have signaled with bold numbers the best fit correlations in the columns assigned to the Higuchi, first order and zero order models, as described in the paragraphs above.

One interesting feature of the data in Table 1 is that the value of the release constant decreases as the number of polymer layers increases, which indicates a substantial reduction in the cumulative release rate when the polyelectrolytes are added to the system (Figure 6a). Thus, coating with alternating polyelectrolyte layers provide a densely packed polymer network due to strong electrostatic interactions, thereby reducing the diflunisal release rate from the oil-core. This phenomenon has been reported by other authors in other systems [15].

## 4. Conclusions

Multilayer polymeric nanocapsules were synthesized by a layer-by-layer assembly approach with good stability and size (~300 nm) suitable for interactions with biological systems at mesoscopic level; therefore, for drug delivery applications. The ζ-potential data collected verified the stability and the successful formation of polyelectrolyte assemblies on the liquid NE colloidal template due to the strong electrostatic repulsive forces between them (less than −30 mV or more than +45 mV) and the surface charge inversion obtained after each deposited polyelectrolyte layer. FTIR and XEDS analyses also proved the presence of characteristic elements and functional groups of each polymer after they were deposited. Spherical and smaller particles were observed by transmission electron microscopy and scanning electron microscopy compared to the hydrodynamic average diameter obtained by DLS analysis of the systems in different sample state. Furthermore, the presence of a liquid oil-core is beneficial in terms of a higher encapsulation efficiency (more than 70%); meanwhile, depending on the number of layers deposited, the mechanism of DF release varies, achieving zero order release of diflunisal from the NE(κ-CAR/CS)_1_-κ-CAR (three layers) and NE(κ-CAR/CS)_2_ (four layers) systems. Biopolymer-coated NE loaded with DF exhibited better release properties than bare NE during cumulative release studies at pH 7.4. These properties could be extremely useful to incorporate lipophilic substances in the food, agricultural or pharmaceutical industry.

## Figures and Tables

**Table 1:** Model parameters for DF-NE(κ-CAR/CS)*_x_* systems at 37 °C and pH 7.4.

System	Korsmeyer-Peppas			Higuchi		First Order		Zero Order	
	*k_KP_* × 10^2^ (min^−*n*^)	*n*	*R* ^2^	*k_H_* × 10^2^ (min^−0.5^)	*R* ^2^	*k*_1_ × 10^2^ (min^−1^)	*R* ^2^	*k*_0_ × 10^2^ (min^−1^)	*R* ^2^
NE	7.5 ± 0.4	0.56 ± 0.02	0.9883	9.2 ± 0.1	**0.9801**	2.6 ± 0.1	0.9177	1.7 ± 0.1	0.6898
NE-κ-CAR	3.8 ± 0.1	0.74 ± 0.01	0.9754	8.4 ± 0.1	0.8973	2.3 ± 0.1	**0.9879**	1.6 ± 0.1	0.9190
NE(κ-CAR/CS)_1_	2.2 ± 0.1	0.89 ± 0.01	0.9760	7.9 ± 0.3	0.8271	2.1 ± 0.1	**0.9791**	1.5 ± 0.1	0.9729
NE(κ-CAR/CS)_1_-κ-CAR	1.4 ± 0.2	0.97 ± 0.02	0.9877	7.1 ± 0.1	0.8006	1.7 ± 0.1	0.9717	1.3 ± 0.1	**0.9893**
NE(κ-CAR/CS)_2_	1.0 ± 0.1	0.99 ± 0.04	0.9748	6.2 ± 0.5	0.7648	Na	-	1.0 ± 0.1	**0.9793**

Bold type indicates the best fitting model after studying the Korsmeyer-Peppas equation; *R*^2^ is the determination coefficient.

## Supplementary Materials

The following are available online at http://www.mdpi.com/2073-4360/10/7/760/s1, Figure S1: AFM images of (**a**) NE and (**b**) NE(κ-CAR/CS)_2_, Figure S2: FTIR spectra of DF, NE and DF-loaded NE.
