# Supplementary material for: Drug Release Properties of Diflunisal from Layer-By-Layer Self-Assembled κ-Carrageenan/Chitosan Nanocapsules: Effect of Deposited Layers
Source: Polymers (Basel). 2018 Jul 10;10(7):760. doi: 10.3390/polym10070760 (PMC6403737; doi:10.3390/polym10070760)
Supplement: Supplementary file 1 [file polymers-10-00760-s001.pdf]

Supplementary Materials:

Drug Release Properties of Diflunisal from Layer-by-layer Self-assembled κ-Carrageenan/Chitosan Nanocapsules: Effect of Deposited Layers

Sarai Rochín-Wong [1], Aarón Rosas-Durazo [2], Paul Zavala-Rivera [3], Amir Maldonado [4], María Elisa Martínez-Barbosa [1], Itziar Vélaz[5,*], and Judith Tánori [1,*]

[1] Departamento de Investigación en Polímeros y Materiales, Universidad de Sonora, 83000 Hermosillo, Sonora, México; sarai.rochin@unison.mx (S.R.-W.); memartinez@polimeros.uson.mx (M.E.M.-B.)
[2] Rubio Pharma y Asociados S.A. de C.V., 83210 Hermosillo, Sonora, México; aaron.rosas@rubiopharma.com
[3] Departamento de Ingeniería Química y Metalurgia, Universidad de Sonora, 83000 Hermosillo, Sonora, México; paul.zavala@unison.mx
[4] Departamento de Física, Universidad de Sonora, 83000 Hermosillo, Sonora, México; maldona@guaymas.uson.mx
[5] Departamento de Química, Facultad de Ciencias, Universidad de Navarra, 31080 Pamplona, Navarra, Spain

* Correspondence: itzvelaz@unav.es; Tel.: +34 948425600 (I.V.); jtanori@unison.mx; Tel.: +526622592161 (J.T.)

Received: date; Accepted: date; Published: date

1. Materials and Methods

1.1. Atomic force microscopy

AFM images were obtained (AFM; Veeco diInnova, Bruker) in the tapping mode. AFM imaging was performed with a spring contact of k = 0.06 N m^{-1} using a nanoprobe cantilever made of silicon nitride (SNL-10 Veeco, Bruker). The scanning speed and resolution were 1.0 Hz and 256 samples per line, respectively. Measurements were performed on dry samples (24 h vacuum desiccator), at room temperature, and on a vibration isolation table (TMC, AMETEK).

2. Results and Discussion

2.1. Atomic force microscopy images

AFM images of both NE (Figure S1a) and NE(κ-CAR/CS)$_2$ oil-core nanocapsules (Figure S1b) can be seen in Figure S1. In general, smaller spherical particles are observed compared to the hydrodynamic diameter obtained by photon correlation spectroscopy (Figure 2). Probably, the drying of the samples and the scanning force acting on the nanocapsule surface during the AFM analysis induced this shrinkage [1]. The height of ~20 nm observed in the systems indicates flattening during the analysis [2].

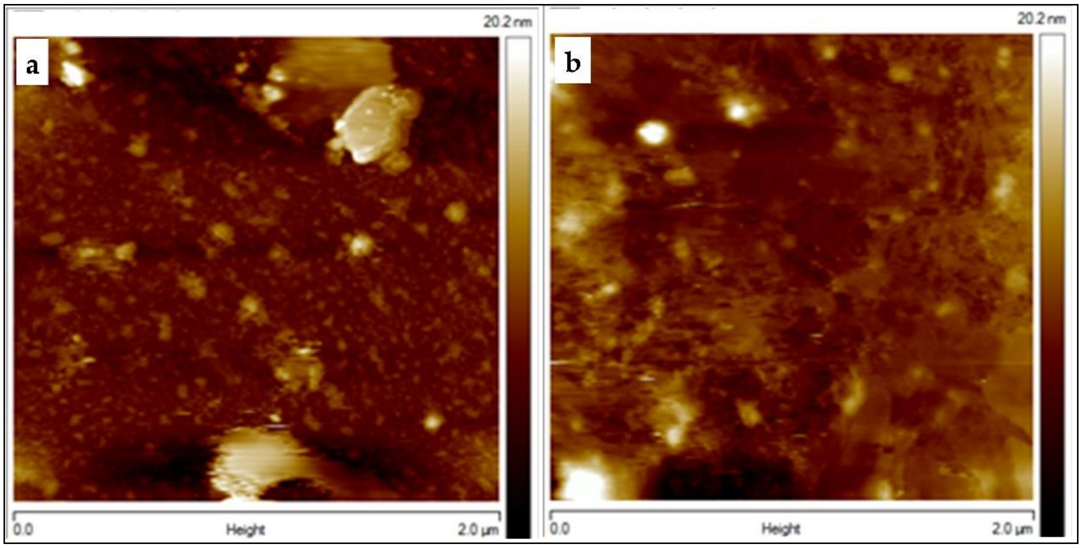

Figure S1. AFM images of (a) NE and (b) NE(κ-CAR/CS)$_2$.

2.2. Fourier transform infrared spectroscopy of DF-loaded nanoemulsion.

The FTIR spectra for diflunisal (DF), nanoemulsion (NE), and nanoemulsion loaded with diflunisal (DF-NE) are shown in Figure S2 in the range of 3500-500 cm^{-1}. The anti-inflammatory DF presented characteristic peaks. The aromatic C-H stretch interfered with the O-H band, and an area of multiple peaks was observed of approximately between 3400-2800 cm^{-1}. On the other hand, at 1670 cm^{-1} associated with the CO stretch in the carboxylic group and C-F stretch at 1265 cm^{-1} [3]. The bands observed in the low frequency region (1600-600 cm^{-1}) in the DF-NE spectrum represent peaks attributed to both diflunisal and NE which show the presence of the drug in the system indicating no interaction.

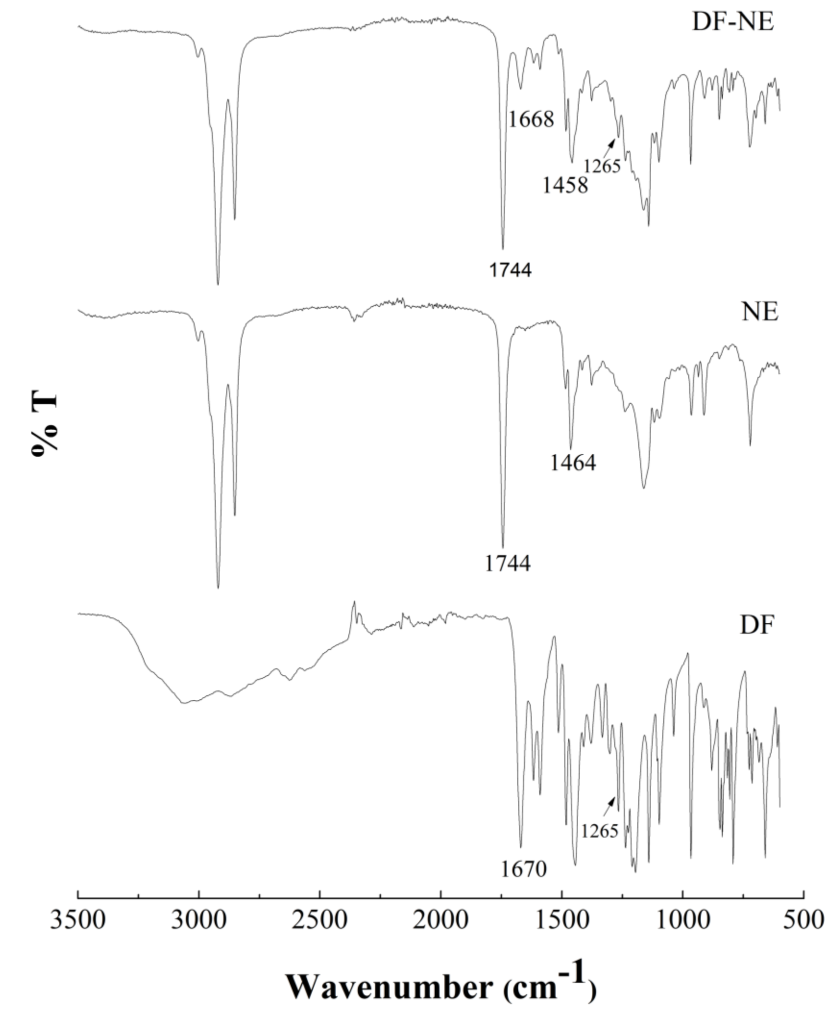

Figure S2. FTIR spectra of DF, NE, and DF-loaded NE.

References

1. Lulevich, V. V.; Radtchenko, I. L.; Sukhorukov, G. B.; Vinogradova, O. I. Deformation properties of nanoadhesive polyelectrolyte microcapsules studied with the atomic force microscope. *J. Phys. Chem. B* **2003**, *107*, 2735–2740, doi:10.1021/jp026927y.

2. Oliveira, L. T.; de Paula, M. A.; Roatt, B. M.; Garcia, G. M.; Silva, L. S. B.; Reis, A. B.; de Paula, C. S.; Vilela, J. M. C.; Andrade, M. S.; Pound-Lana, G.; Mosqueira, V. C. F. Impact of dose and surface features on plasmatic and liver concentrations of biodegradable polymeric nanocapsules. *Eur. J. Pharm. Sci.* **2017**, *105*, 19–32, doi:10.1016/j.ejps.2017.04.017.

3. Kaur, A.; Goindi, S.; Katare, O. P. Thermal analysis and quantitative characterization of compatibility between diflunisal and lipid excipients as raw materials for development of solid lipid nanoparticles. *Thermochim. Acta* **2016**, *643*, 23–32, doi:10.1016/j.tca.2016.09.014.
